# Interchromosomal contacts between regulatory regions trigger stable transgenerational epigenetic inheritance in *Drosophila*

**DOI:** 10.1016/j.molcel.2024.11.021

**Published:** 2024-12-11

**Authors:** Maximilian H. Fitz-James, Gonzalo Sabarís, Peter Sarkies, Frédéric Bantignies, Giacomo Cavalli

**Affiliations:** 1 Institute of Human Genetics, CNRS and University of Montpellier, 141 Rue de la Cardonille, 34094 Montpellier, France; 2 Department of Biochemistry, University of Oxford, South Parks Road, Oxford OX1 3QU, UK

## Abstract

Non-genetic information can be inherited across generations in a process known as transgenerational epigenetic inheritance (TEI). In *Drosophila*, hemizygosity of the *Fab-7* regulatory element triggers inheritance of the histone mark H3K27me3 at a homologous locus on another chromosome, resulting in heritable epigenetic differences in eye color. Here, by mutating transcription factor binding sites within the *Fab-7* element, we demonstrate the importance of the proteins pleiohomeotic and GAGA factor in the establishment and maintenance of TEI. We show that these proteins function by recruiting the polycomb repressive complex 2 and by mediating interchromosomal chromatin contacts between *Fab-7* and its homologous locus, respectively. Using an *in vivo* synthetic biology system to induce them, we then show that chromatin contacts alone can establish TEI, providing a mechanism by which hemizygosity of one locus can establish epigenetic memory at another distant locus in *trans* through chromatin contacts.

## Graphical abstract

**Graphical abstract:**
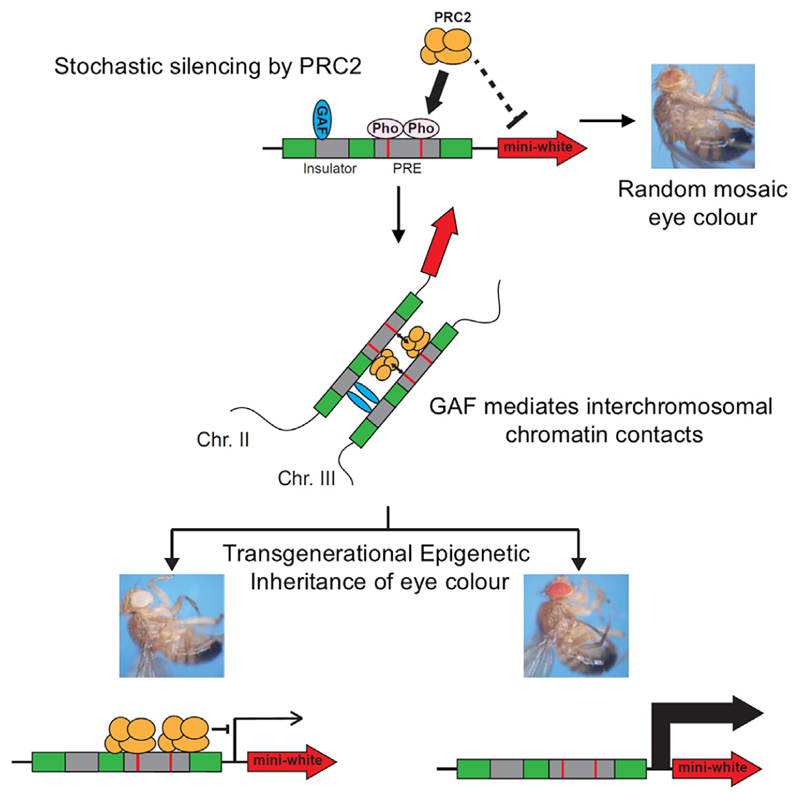

## Introduction

Epigenetic information has long been known to be a major factor in the regulation of gene expression.^1^ Whether such information can be transmitted across generations in various organisms has been a more elusive question, made difficult by the potential for genetic factors to confound experiments on heredity.^2,3^ Through careful experimentation in model organisms, recent work has now demonstrated that such transgenerational epigenetic inheritance (TEI) does occur in a variety of organisms.^4,5^ In addition, although more work is required, the molecular mechanisms underlying these instances of inheritance have begun to be described.^6^ Many well-known epigenetic regulators of gene expression have been implicated in different cases of TEI, including non-coding RNAs,^7–12^ DNA methylation,^13–15^ and histone modifications,^12,16–19^ although a broader definition of TEI may also include less-typical sources of non-genetic information, such as prions,^20^ three-dimensional (3D) chromatin organization,^19^ RNA methylation,^21,22^ and transcription factor binding,^23^ which may act as secondary signals in some cases.

Just as genes and their allelic variants are the basis of genetic variation, so are “epialleles”—the basic units of heritable epigenetic change. Similarly, whereas mutation is the means by which genetic variation arises, “epimutation” describes the appearance of a heritable change in epigenetic information that gives rise to an epiallele. Epimutation provides an alternative source of heritable variation that differs from genetic mutation in that it has the potential to be more rapid, targeted, and reversible, allowing for fast adaptation to a fluctuating environment.^24^ Nonetheless, the underlying causes of epimutations and the mechanism by which they arise remain unclear and likely vary between organisms.

Given the complexity of TEI in higher eukaryotes, model systems that can be easily manipulated and allow one to both effectively track heritable phenotypes and analyze the underlying molecular events at play are critically important in establishing mechanistic principles. One such example of TEI occurs in *Drosophila melanogaster* in a transgenic line called “Fab2L,” involving a transgene that drives the expression of the eye pigmentation gene *mini-white*.^25^ Flies of the Fab2L line exhibit a stochastic phenotype, manifesting as mosaicism of pigmentation in the adult eye. Though it is initially random, a memory of eye color can be established by a transient genetic perturbation,^19^ which can then be maintained epigenetically for countless generations following the initial trigger. Although this represents a clear case of TEI, the mechanism by which this epigenetic memory is established remains unclear, making Fab2L a valuable model system to study the means by which epimutations arise. Here, we investigate the mechanistic basis for the establishment of TEI at the Fab2L locus. We show that it is mediated by two key regulatory regions through the binding of the transcription factors pleiohomeotic (Pho) and GAGA factor (GAF).

We show that Pho is the primary recruiter of the polycomb repressive complex 2 (PRC2) to the transgene, leading to deposition of H3K27me3, whereas GAF promotes interchromosomal chromatin contacts between the transgene and a homologous region elsewhere in the genome. Using an *in vivo* synthetic biology system, we artificially recapitulate these contacts to demonstrate that chromatin contacts alone are sufficient to induce TEI in *Drosophila*, providing a mechanism whereby genetic perturbation of one locus can trigger TEI at another in *trans* through chromatin interactions.

## Results

### Binding of GAF and Pho is responsible for epigenetic variability at the Fab2L transgene

**Figure 1:**
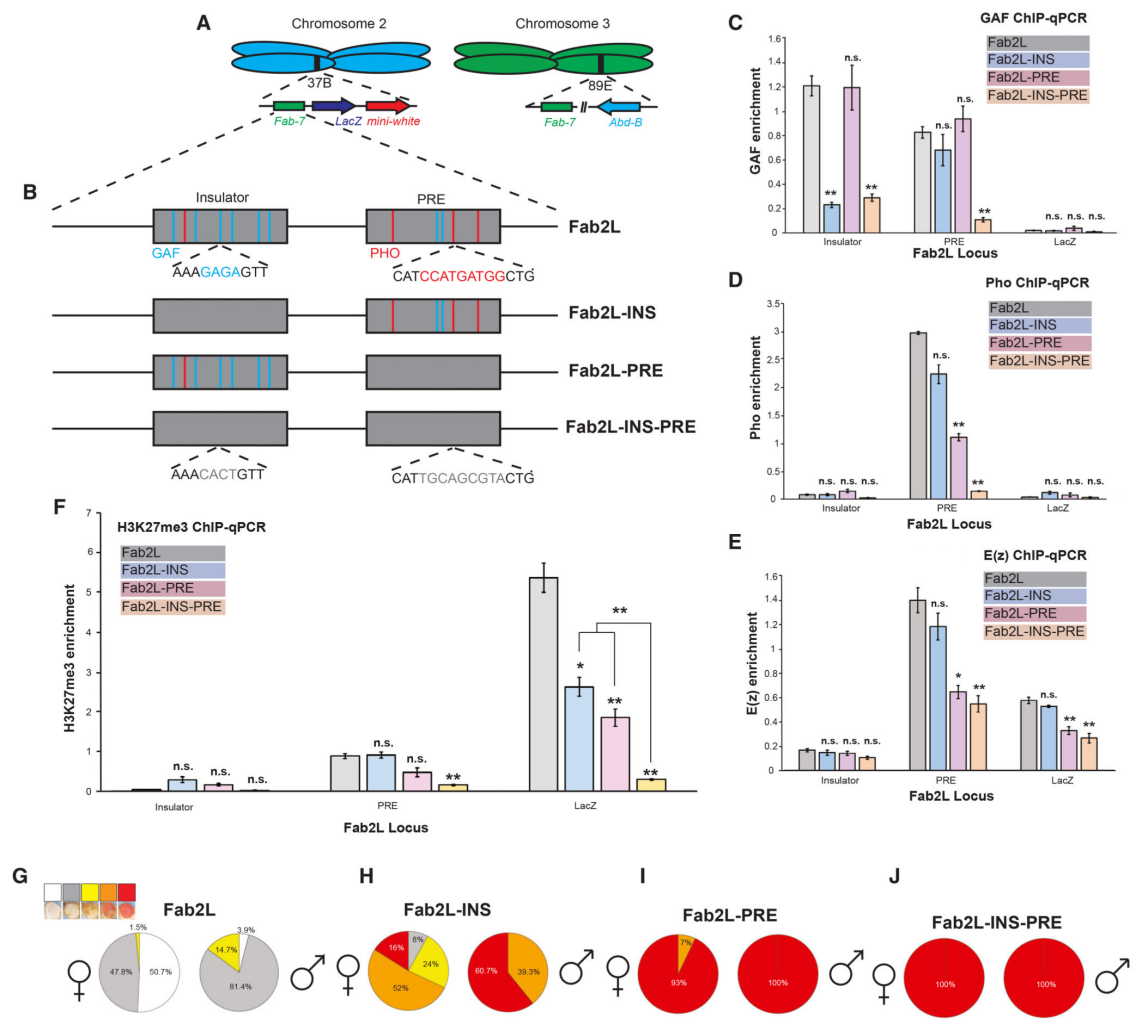
Mutation of GAF and Pho binding sites decreases epigenetic variability of the Fab2L transgene (A) Schematic representation of the Fab2L transgene at its insertion site at cytological position 37B on chromosome 2, alongside the homologous *Fab-7* region on chromosome 3. (B) Illustration of the *Fab-7* element with important subdomains and transcription factor binding sites in wild-type and mutated versions of the Fab2L transgene. (C–F) ChIP-qPCR assays performed in embryos of the indicated genotypes at regions within the Fab2L transgene. Error bars represent ± standard error from the mean (SEM) of three independent repeats. Samples were normalized to engrailed as a positive control and compared with wild-type Fab2L or between each other by the t test (*p < 0.05, **p < 0.01, n.s., not significant). (G–J) Phenotypic classification of eye color in female and male adults of the indicated genotypes. Flies were sorted into five classes on the basis of eye color, representing the number of pigmented ommatidia: class 1 = 0%; class 2 = 1%–10%; class 3 = 10%–75%; class 4 = 75%–99%; class 5 = 100%. See also Figure S1.

The *Drosophila* Fab2L line carries a single-copy 12.4-kb transgene inserted into chromosome arm 2L at cytogenetic position 37B.^25,26^ This transgene contains the reporter genes *LacZ* and *mini-white*, under the control of the *Fab-7* element. *Fab-7* is a well-studied regulatory region of the bithorax complex (BXC) on chromosome 3, where it regulates the expression of the Hox gene *Abdominal B* (*Abd-B*).^27–29^ Importantly, the Fab2L line therefore contains two versions of the *Fab-7* element in its genome, one at its endogenous location on chromosome 3 and one inserted ectopically within the transgene on chromosome 2 (Figure 1A).

The *mini-white* reporter gene, which controls red pigment deposition in the eye, is not expressed uniformly in Fab2L flies, giving a mosaic pattern to eye pigmentation, with some ommatidia showing strong *mini-white* expression and others strong repression within the same individual (Figures S1A and S1B). Stochastic binding of the PRC2, which deposits the repressive histone mark H3K27me3, has been suggested as an explanation for the variability of *mini-white* expression in transgenes carrying polycomb-bound elements. This mosaic eye pattern therefore represents a very evident and visible instance of epigenetic variation in the absence of any underlying genetic change.^19^

*Fab-7* contains within it both an insulator region, which in its endogenous state prevents mis-regulation of *Abd-B* by adjacent regulatory regions in the wrong body segments, and a polycomb response element (PRE), which recruits either polycomb group (PcG) or trithorax group (TrxG) proteins to maintain the pattern of *Abd-B* expression established early in development. These regions contain several consensus sequence motifs for the DNA-binding proteins Pho and GAF (Figure 1B). Pho is the primary recruiter of PRC2,^30^ a role consistent with the presence of its binding sites, predominantly in the PRE region (3 out of 4 total sites). The function of GAF is more complex but includes regulation of gene expression through interaction with PcG and TrxG as well as an insulator function.^31–34^ In the *Fab-7* element, 6 out of 9 GAF sites are located in the insulator. We sought to further investigate the role of these proteins, as well as the two *Fab-7* subdomains, in the establishment and inheritance of epigenetic memory at the Fab2L locus.

We generated three transgenic lines mutating all GAF and Pho sequence motifs within either the insulator (Fab2L-INS), the PRE (Fab2L-PRE), or both (Fab2L-INS-PRE) in the Fab2L transgene (Figure 1B). Fab2L-INS showed a significant decrease in GAF binding to the insulator, although Pho binding was little affected as was GAF binding to the PRE. Conversely, Fab2L-PRE exhibited significantly decreased Pho binding at the PRE only and did not affect GAF binding at either the insulator or the PRE (Figures 1C and 1D). Consistent with the profile of Pho binding, PRC2 binding was largely unaffected in Fab2L-INS but significantly decreased in Fab2L-PRE, both at the PRE itself and at the downstream LacZ region (Figure 1E). Mutation of both regions together showed clear additive effects. Indeed, in the Fab2L-INS-PRE line, binding of GAF and Pho is decreased at both the insulator and PRE and, in most cases, to a significantly greater extent than that in either of the single mutants (Figures 1C and 1D), although recruitment of PRC2 was not significantly different from Fab2L-PRE (Figure 1E). Taken together, these results suggest that the insulator and PRE regions of *Fab-7* act cooperatively to recruit GAF and Pho to the Fab2L transgene, although the PRE may play the greater role in the subsequent recruitment of PRC2.

We then analyzed the downstream effects of altered GAF and Pho recruitment in these mutants. All three mutant lines had significantly decreased levels of H3K27me3 at the LacZ region of the Fab2L transgene (where H3K27me3 levels are highest in the wild type [WT]), with the decrease being much more pronounced in the double-mutant Fab2L-INS-PRE line (Figure 1F). Decreased H3K27me3 was also observed at the PRE in the Fab2L-INS-PRE mutants. These changes in chromatin translated to phenotypic effects on adult eye color. Indeed, all three mutant lines displayed shifts toward red eye color compared with naive Fab2L (Figures 1G–1J and S1). The shift in Fab2L-INS was milder than for the other two lines, with approximately 16% of females and 61% of males exhibiting fully red eyes (Figures 1H, S1C, and S1D). In contrast, almost all Fab2L-PRE and Fab2L-INS-PRE flies of both sexes exhibited fully red eyes. Interestingly, however, although all Fab2L-INS-PRE individuals had uniform red eyes (Figures 1J, S1G, and S1H), Fab2L-PRE retained some stochasticity, with around 7% of females possessing at least some white ommatidia (Figures 1I and S1E). These results reinforce the idea that the insulator and PRE together are responsible for the epigenetic and phenotypic variability of the Fab2L fly line.

### The insulator and PRE regions of *Fab-7* are individually sufficient to mediate TEI

In WT flies carrying the Fab2L transgene, epigenetic differences in expression between individuals are not initially inherited transgenerationally. Indeed, when we applied repeated selection and crossing of the most extreme individuals in the population of an unmanipulated Fab2L line over ten generations, we did not obtain any significant differences in eye color across the population (Figures S2A–S2C). Reproducing a previous experiment, we crossed this “naive” Fab2L line with another Fab2L line bearing a homozygous deletion of the endogenous *Fab-7* to produce F1 individuals homozygous for the transgene and hemizygous for the endogenous *Fab-7* (Figure S2D). As previously reported, hemizygosity of the endogenous *Fab-7* locus establishes transgenerational epigenetic memory at the transgene, such that reconstituting the Fab2L genotype in the F2 resulted in a line that is genetically identical to the P0 Fab2L but in which TEI is now possible.^19^ Indeed, selection of this line over 10 generations resulted in either red or white “epilines”: populations of flies with a significant proportion of individuals with monochrome eye color, i.e., with 100% of their ommatidia either pigmented or unpigmented (Figures S2E and S2F). We also found that other crossing schemes that induced *Fab-7* hemizygosity for one or two generations were able to trigger TEI in Fab2L (Figures S2G–S2P).

**Figure 2:**
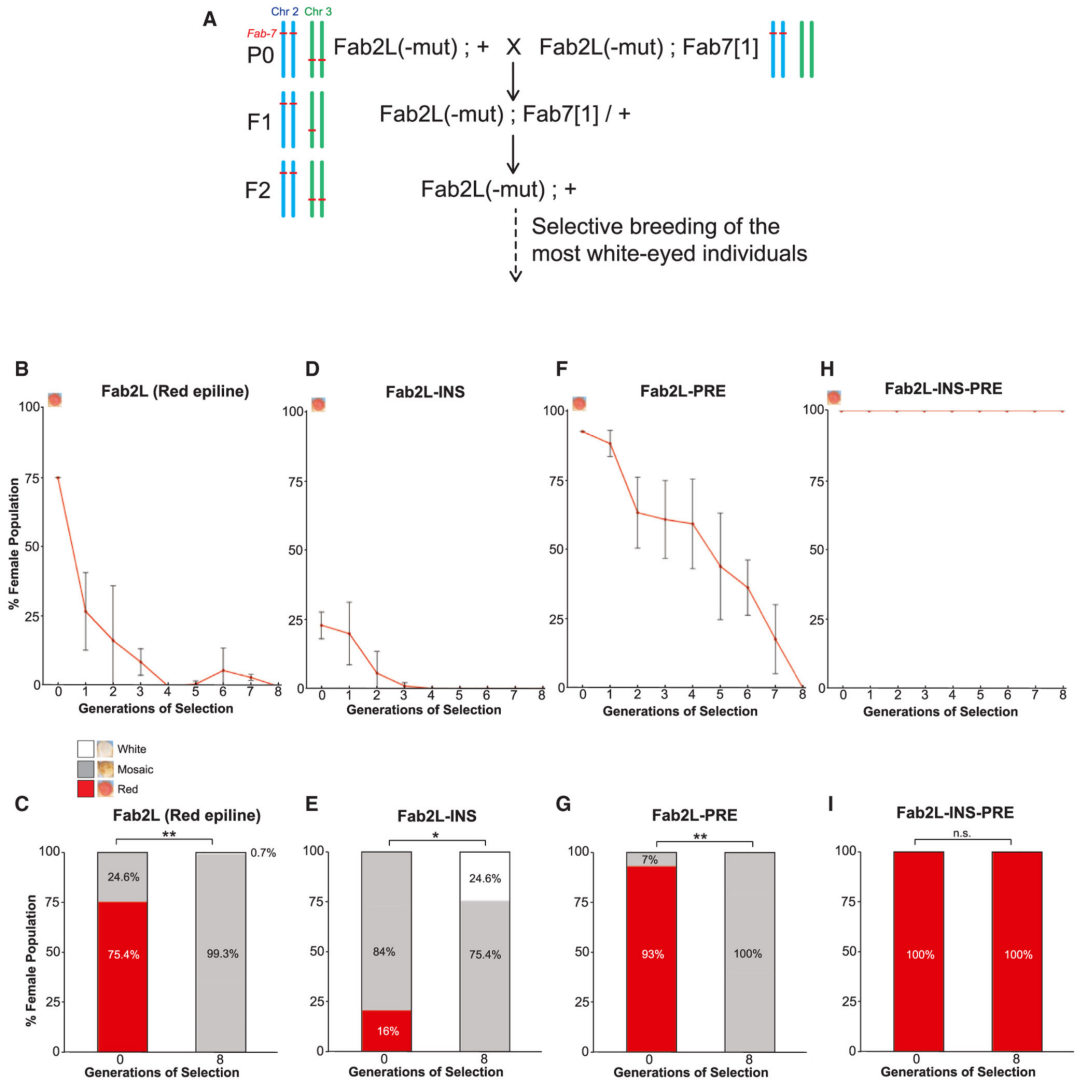
Epigenetic inheritance of eye color is abrogated in the absence of GAF and Pho binding to the Fab2L transgene (A) Crossing scheme for the triggering of TEI at wild-type and mutant versions of Fab2L, with diagrammatic representation of the copy number of the *Fab-7* element on chromosomes 2 and 3. See also Figure S2. (B, D, F, and H) Results of selection for the most white-eyed flies in each generation beyond the F2 of the crossing scheme. Curves represent the percentage of class 5 females in the population across generations. Error bars are ± standard deviation (SD) of 3 independent repeats. (C, E, G, and I) Bar graphs representing the phenotypic distribution of eye color within the female population in the first and last generations of selection, averaged over 3 repeats. The average percentage of class 5 (fully red) individuals was compared using the t test (*p < 0.05, **p < 0.01, n.s. = not significant). See also Figure S3.

Given the altered phenotype of the mutant Fab2L transgenic lines, we asked whether these mutations also interfered with the ability of the Fab2L transgene to maintain a memory of its epigenetic state across generations by TEI. Due to the shift toward red eyes in the naive Fab2L-INS, Fab2L-PRE, and Fab2L-INS-PRE lines, selection toward red eyes would not be informative. We therefore performed a transgenerational epigenetic selection experiment to determine whether these mutant lines could be selected toward a more repressed, white-eyed phenotype than the naive population. Just as with WT Fab2L (Figure S2D), we introduced a single generation of *Fab-7* hemizygosity while leaving the mutant versions of the Fab2L transgene unmanipulated (Figure 2A). We then reconstituted the parental genotype, homozygous for the endogenous *Fab-7*, and selectively bred the most white-eyed individuals over subsequent generations. As a control, we used a WT Fab2L line that had previously been selected for a red-eyed phenotype. The Fab2L, Fab2L-INS, and Fab2L-PRE, which all showed a greater or lesser degree of variability in their starting populations, were receptive to selection, showing a clear and gradual shift toward whiter eyes in both females and males over the generations (Figures 2B–2G and S3A–S3F). In the case of Fab2L-INS, the appearance of some individuals with fully white eyes was even observed (Figures 2E and S3D), consistent with the less-extreme de-repression observed in this line compared with the other mutants. In contrast, the Fab2L-INS-PRE line displayed no variation at any point during the experiment, with all individuals of both sexes maintaining a uniform red eye color (Figures 2H, 2I, S3G, and S3H). As expected, some pre-existing degree of polycomb binding and epigenetic variation at the Fab2L transgene is therefore a prerequisite for TEI. However, these results also demonstrate that the insulator or PRE regions alone are still able to mediate TEI, indicating that they are at least partially redundant in maintaining an epigenetic memory at the transgenic *Fab-7* element in the Fab2L line.

### The insulator and PRE regions of *Fab-7* can mediate horizontal transmission of a repressed epigenetic state through paramutation

**Figure 3:**
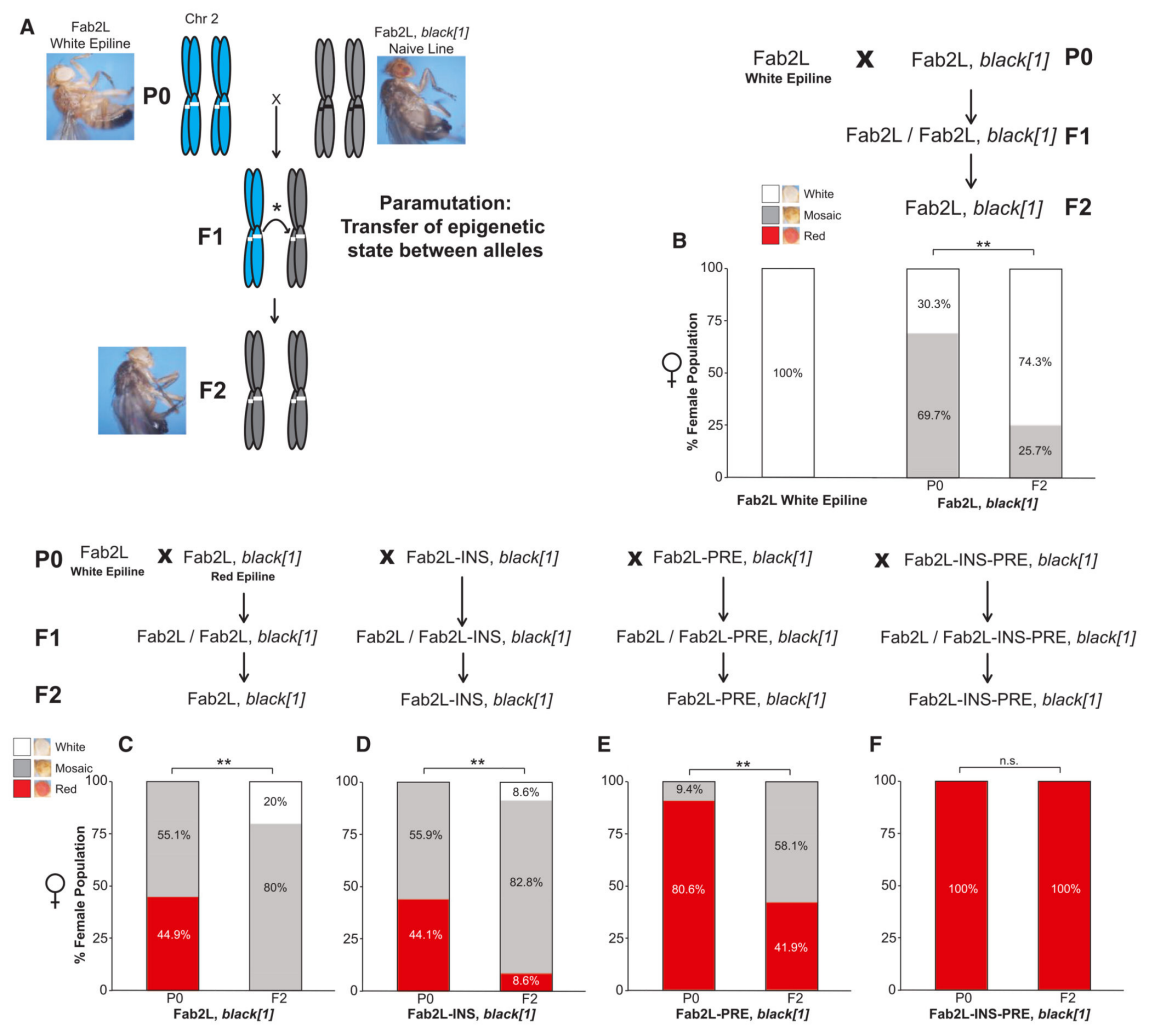
The insulator and PRE regions are required for Fab2L to acquire a repressed epigenetic state through paramutation (A) Illustration of the paramutation crossing scheme for acquisition of a repressed epigenetic state by a naive Fab2L allele from an established epiallele in *trans*. The presence of a *black[1]* marker linked to Fab2L allows for the identification of F2 individuals carrying two copies of the transgene from the naive parent (gray chromosomes) rather than from the epiline parent (blue chromosomes). See also Figure S4. (B–F) Paramutation crossing schemes and phenotypic distribution of the populations with the indicated genotypes before and after the paramutation cross. Bar graphs represent the phenotypic distribution of eye color within the female population. The percentage of class 1 (for B) or class 5 (for C–F) individuals was compared using Fisher’s exact test (*p < 0.05, **p < 0.01, n.s., not significant). See also Figure S5.

The Fab2L transgene is not only able to acquire an altered epigenetic state by selection over generations but also can do so in a single generation by the process of “paramutation.”^19^ Rather than referring to a specific mechanism, paramutation denotes a type of non-mendelian inheritance whereby an epigenetic state is transmitted in *trans* between two homologous alleles. Paramutation can occur through different molecular processes and has been described in many organisms, including *Drosophila*.^35–38^ In the Fab2L line, crossing a naive Fab2L with an established Fab2L epiline (either white or red-eyed) results in the acquisition of the altered epigenetic state of the epiline allele by the naive allele. This phenomenon can be tracked by the use of a *black[1]* marker allele, closely linked to the Fab2L transgene, such that F2 individuals that have inherited both copies of Fab2L from the naive parent can be determined with high probability (Figures 3A and S4A). Although these F2 flies possess the genetic material of the naive P0 population, their epigenetic state more closely resembles that of the epiline with which it was crossed, attesting to the acquisition over this genomic region of a new epigenetic state (Figures 3B, S4B, and S5B). Fab2L epiline identity can thus be transmitted not only vertically (i.e., across generations) but also horizontally between alleles.

To determine whether mutation of the transgene interfered with horizontal transfer of the epigenetic state, we crossed the Fab2L-INS, Fab2L-PRE, and Fab2L-INS-PRE lines with a white-eyed Fab2L epiline to see whether these mutant versions of the Fab2L transgene could acquire a repressed epigenetic state by paramutation. Again, as control, we used a WT Fab2L epiline that had previously acquired a de-repressed, red-eyed epigenetic state. Fab2L, Fab2L-INS, and Fab2L-PRE were able to acquire a more white-eyed phenotype than the naive parental lines (Figures 3C–3E and S5C–S5E). Conversely, all Fab2L-INS-PRE individuals maintained their uniform red coloration of the eyes even after exposure in the F1 of the cross to a repressed epiallele (Figures 3F and S5F). Although lines bearing a WT version of the insulator or PRE of *Fab-7* alone were therefore able to acquire an altered epigenetic state by both selection over generations and paramutation, mutation of both regions together completely prevents acquisition of a repressed epigenetic state by either method. Taken together, these results therefore suggest that the presence of at least one of the two *Fab-7* subdomains (i.e., the insulator or the PRE) is essential, not only to mediate epigenetic variation at the transgenic *Fab-7* but also to acquire and maintain an epigenetic memory across generations.

### GAF mediates chromatin contacts between distant *Fab-7* elements

Our results point to PRC2 and GAF as key factors mediating epigenetic variability, and its inheritance across generations, at the Fab2L transgene. PRC2 has a direct role in regulating the expression of the Fab2L transgene, as the differences in *mini-white* expression and phenotype of the Fab2L epilines correlate with differences in PRC2-deposited H3K27me3 across the transgene.^19^ The role of GAF is less clear, as mutation of the GAF sites in the insulator ultimately had little effect on PRC2 binding (Figure 1E). Intriguingly, among its many functions, GAF has been shown to mediate chromatin contacts between its target genes.^34,39,40^ The 3D organization of chromatin is a major factor in the regulation of gene expression, and polycomb target genes in particular are frequently found to colocalize in the nucleus at so-called “polycomb bodies.”^41^ GAF has been proposed as a major driver of these contacts, especially between distant paralogous genes.^42^ This is highly relevant, as the transgenic and endogenous copies of *Fab-7*, which could be considered distant paralogs, were previously found to form chromatin contacts in the Fab2L line.^19^

**Figure 4:**
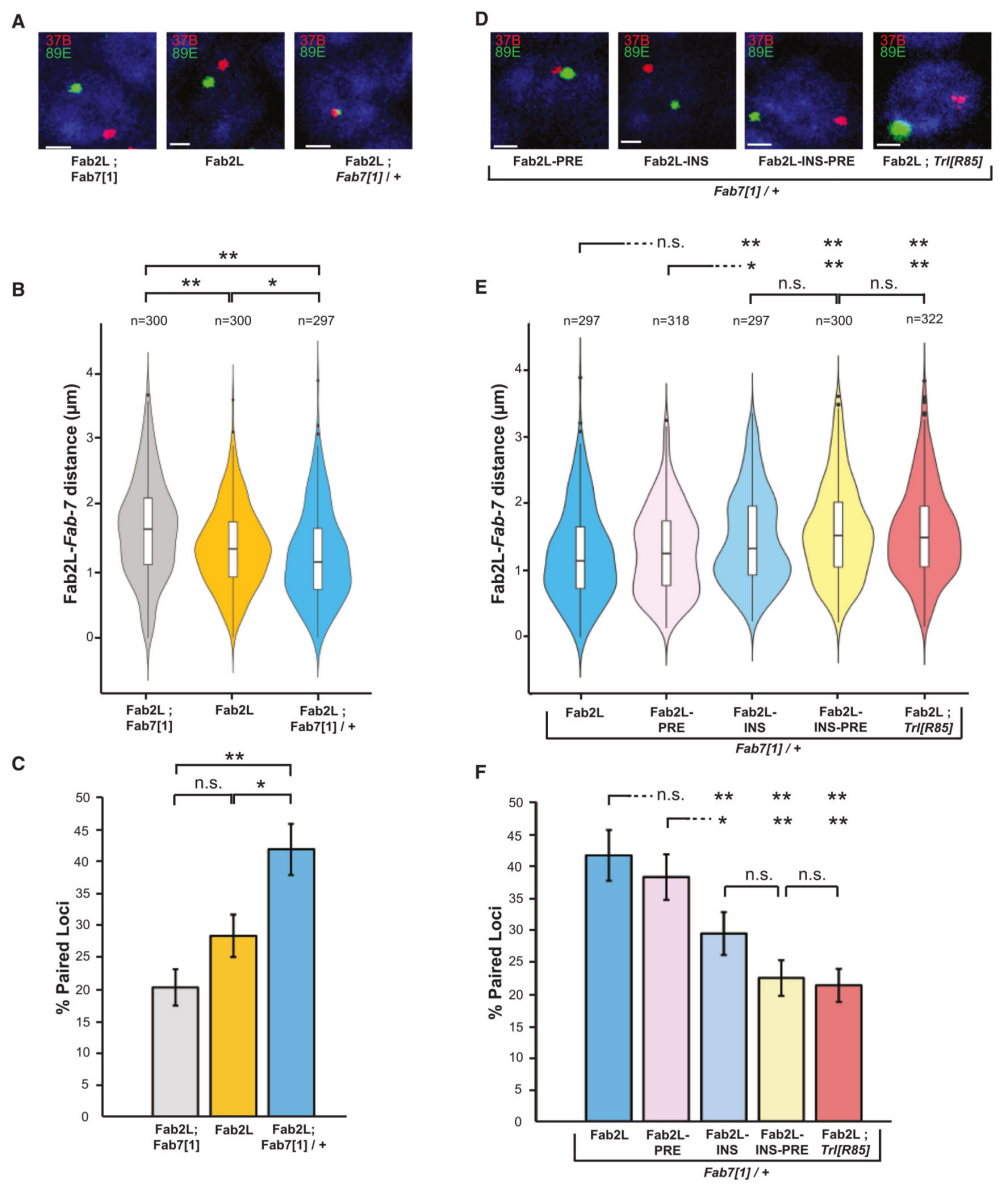
GAF binding to the insulator mediates chromatin contacts between Fab2L and *Fab-7* (A and D) Illustrative micrographs of FISH in embryonic nuclei of the indicated genotypes. Nuclei are stained with DAPI in blue, the loci surrounding the Fab2L transgene (37B) and endogenous *Fab-7* (89E) are stained in red and green, respectively. Scale bars represent 1 mm. See also Figure S6. (B and E) Violin plots representing the distribution of average distance between the 37B (Fab2L) and 89E (*Fab-7*) loci as determined by FISH in the indicated genotypes. Distances were measured in stage 14–15 embryos in T1 and T2 segments. (C and F) Bar graphs representing the percentage of cells from the same FISH assays in which the inter-loci distance was less than 1 mm. Both distance distributions and paired loci percentages were compared by ANOVA (*p < 0.05, **p < 0.01, n.s., not significant).

We performed fluorescence *in situ* hybridization (FISH) to quantify chromatin contacts between the regions surrounding the *Fab-7* elements in Fab2L embryos carrying different copy numbers of the endogenous *Fab-7* (Figures 4A and S6A–S6C). As previously reported, these two regions frequently colocalized in the nuclei of Fab2L embryos but not in Fab2L; *Fab7[1]* embryos, which lack the endogenous *Fab-7*, resulting in a significant decrease in the average distance between the two loci measured across many nuclei (Figure 4B). This showed that chromatin contacts do occur between these loci, dependent on the presence of both *Fab-7* elements. Intriguingly, these Fab2L-*Fab-7* chromatin contacts were even more frequent in a *Fab7[1]*/+ genetic background in which only one copy of the endogenous *Fab-7* is present. Indeed, these embryos hemizygous for *Fab-7* showed a further decrease in average distance between the two loci (Figure 4B), as well as a highly significant increase in the proportion of cells in which the two loci colocalized (Figure 4C).

To investigate the role of the Fab2L subdomains in these chromatin contacts, we extended the FISH analysis to our mutated transgenic lines (Figures 4D and S6D–S6F). Mutation of the PRE in the Fab2L transgene had no significant effect on the chromatin contacts compared with WT Fab2L, measured either by average inter-loci distance (Figure 4E) or proportion of nuclei with colocalized loci (Figure 4F). Conversely, contacts were significantly decreased upon mutation of the insulator—and even further upon mutation of the insulator and PRE together. These results suggest a primary role for the insulator in mediating these chromatin contacts, which we hypothesized was due to the action of GAF. Thus, mutation of the insulator, containing the majority of GAF sites, eliminates the majority of contacts, whereas mutation of all GAF sites across both insulator and PRE completely eliminates contacts.

To test this hypothesis, we measured Fab2L-*Fab-7* distance in embryos containing a WT version of the Fab2L transgene but homozygous for the *Trl^R85^* mutation (Figures 4D and S6G). This mutation consists of a large deletion in the *Trithorax-like* (*Trl*) gene, which encodes GAF, eliminating its functionality.^43^ Though homozygous lethal, *Trl^R85^* flies survive until the larval stage,^44^ allowing for FISH analysis in embryos. Just as in the Fab2L-INS-PRE mutant, these flies showed complete loss of Fab2L-*Fab-7* contacts (Figures 4E and 4F). Taken together, these results indicate that it is the action of GAF, primarily through association with the *Fab-7* insulator, which leads to the establishment of interchromosomal chromatin contacts between the transgenic and endogenous *Fab-7* elements.

### Artificially induced chromatin contacts are sufficient to induce TEI at the Fab2L locus

Although the role of PRC2 in depositing the inherited H3K27me3 mark was clear, the function of these GAF-mediated chromatin contacts in TEI at the Fab2L locus was not. We therefore investigated the potential involvement of chromatin contacts mediated by GAF in different aspects of TEI. Potentially, chromatin contacts might contribute to either maintenance or initiation of TEI. Inter-loci Fab2L-*Fab-7* distance does not differ significantly between white- or red-eyed epilines of Fab2L,^19^ arguing against a contribution of chromatin contacts to the maintenance of epigenetic differences between these epilines. We also found that, although these contacts are robust in late-stage embryos, they are not observable in earlier stages. Indeed, FISH analysis carried out on embryos at different stages across development revealed that the increase in chromatin contacts induced by *Fab-7* hemizygosity only appears at stages 12–13. Even then, high degrees of variation remain between individuals, and frequent colocalization is only reliably observed at stages 14–15 (Figure S7). This late appearance of chromatin contacts indicates that they are unlikely to themselves transmit epigenetic information across generations.

**Figure 5:**
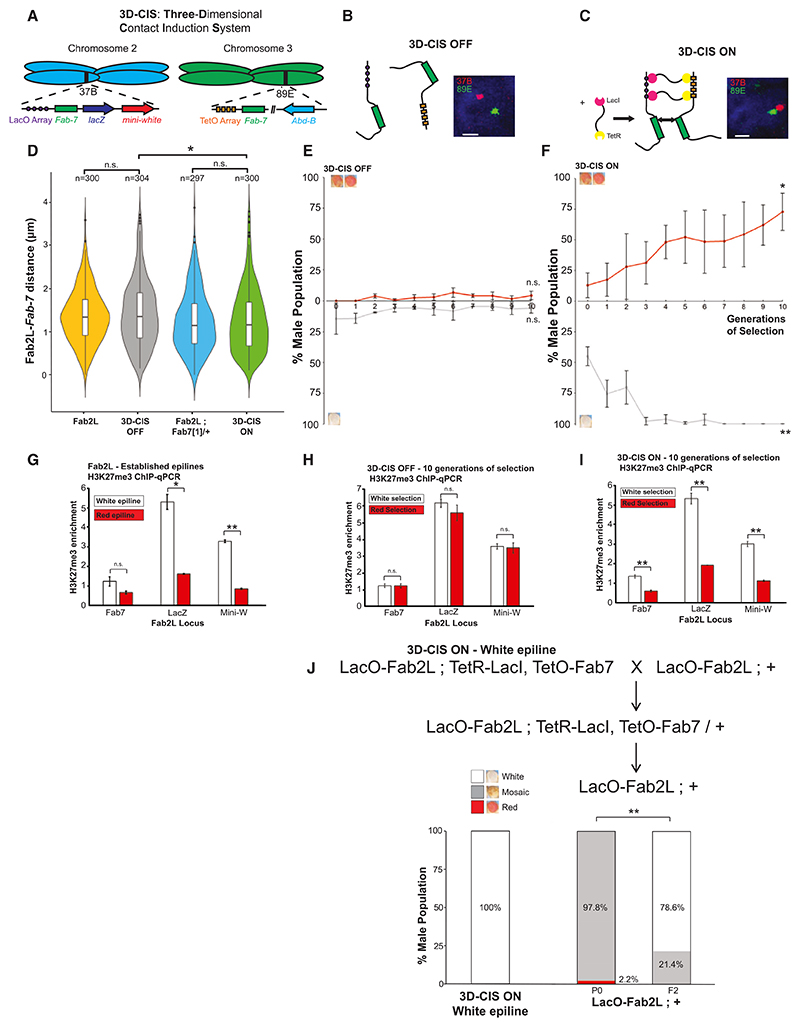
An *in vivo* synthetic biology system promotes interchromosomal contacts between *Fab-7* elements and is sufficient to induce TEI without genetic perturbation (A–C) Schematic representation of 3D-CIS. Arrays of Lac and Tet operons are inserted next to the transgenic and endogenous *Fab-7* elements. Expression of a TetR-LacI fusion protein binding to both arrays promotes contacts between the two loci. Illustrative micrographs represent nuclei from embryos with 3D-CIS in the “OFF” or “ON” state, with FISH highlighting the regions surrounding the *Fab-7* elements. Nuclei are stained with DAPI in blue, the 37B and 89E loci are stained in red and green, respectively. Scale bars represent 1 mm. See also Figures S8 and S9. (D) Violin plots representing the distance distributions of the 37B and 89E regions surrounding the two *Fab-7* elements as determined by FISH in the indicated genotypes. Distances were measured in stage 14–15 embryos in T1 and T2 segments. Distributions were compared by ANOVA (*p < 0.05, **p < 0.01, n.s., not significant). (E and F) Results of selection for the most white or red-eyed flies in each generation of 3D-CIS flies in either the “OFF” or “ON” state. Curves represent the percentage of males of class 1 or class 4 + 5 in the population across generations. Error bars are ± SD of 3 independent repeats. Averages in the final generation were compared with those of the starting generation by the t test (*p < 0.05, **p < 0.01, n.s., not significant). See also Figures S10 and S11. (G–I) ChIP-qPCR assays against H3K27me3 performed in embryos of the indicated genotypes after at least 10 generations of selection toward white or red epiallele identity, at regions within the Fab2L transgene. Error bars represent ± SEM of three independent repeats. Samples were normalized to engrailed and compared by the t test (*p < 0.05, **p < 0.01, n.s., not significant). (J) Paramutation crossing schemes and phenotypic distribution of the populations with the indicated genotypes before and after the paramutation cross. Bar graphs represent the phenotypic distribution of the eye color within the male population. The percentage of class 1 individuals was compared using Fisher’s exact test (**p < 0.01). See also Figure S12.

An alternative hypothesis is that chromatin contacts contribute to initiation of TEI. Supporting this, the increase in Fab2L-*Fab-7* chromatin contacts in Fab2L; *Fab7[1]*/+ individuals (Figures 4A–4C), correlates with the triggering of TEI in genetic crosses (Figure S2). We therefore wished to explore this correlation in greater detail. To directly investigate the role of chromatin contacts in the triggering of TEI, we developed an *in vivo* system to induce inter-chromosomal contacts between the two regions of interest without recourse to any genetic perturbation. We dubbed this system “3D contact induction system” or “3D-CIS,” for its ability to bring two distant loci in proximity (Figures 5A and S8). To create this system, we inserted arrays of Lac or Tet operators adjacent to the transgenic and endogenous *Fab-7* elements, respectively. Aside from the addition of these arrays, this line has the same genotype as the Fab2L line and has a similar average distance between the two loci (Figures 5B, 5D, and S9A). However, activation of the system by introducing a TetR-LacI fusion protein that binds to both arrays (Figures S8E and S8F) results in anchoring of the two *Fab-7* elements to each other (Figure 5C). This anchoring leads to a decrease in the average Fab2L-*Fab-7* distance to a level comparable with the hemizygous Fab2L; *Fab7 [1]*/+ in which TEI is established (Figures 5D and S9B). Importantly, at no point are either the transgenic or endogenous *Fab-7* in a hemizygous state (Figures S8A and S8B). 3D-CIS therefore allows us to investigate the effect of increasing chromatin contacts between the two *Fab-7* elements in the absence of any genetic perturbation.

Just as with Fab2L in the absence of genetic perturbation (Figures S2A and S2B), selection of the 3D-CIS line in the “OFF” state over several generations did not result in any change in eye color across the population toward either white or red eyes (Figures 5B and 5E; for the sake of clarity only male eye color data are presented hereafter, as females followed similar trends). After ten generations of selection, these lines also exhibited no difference in H3K27me3 levels between each other (Figure 5H). However, activation of 3D-CIS, by introduction of the TetR-LacI fusion protein and thus an increase in contacts between the Fab2L transgene and endogenous *Fab-7*, was able to establish TEI, such that selection over subsequent generations resulted in both white and red epilines (Figures 5C and 5F). These epilines also had significant differences in H3K27me3 levels between them (Figure 5I), reminiscent of the differences between Fab2L epilines obtained by selection after transient hemizygosity of *Fab-7* (Figure 5G). These results demonstrate that chromatin contacts alone, in the absence of any genetic perturbation, are sufficient to induce TEI at the Fab2L transgene. As further controls, we generated two more lines expressing either a LacI-LacI or a TetR-TetR fusion protein as part of 3D-CIS (Figures S8C and S8D). These lines were also unable to trigger TEI (Figure S10), showing that triggering was not due to expression of a fusion protein or its binding to either array singly but conclusively resulted from the binding of the fusion protein to both arrays in tandem.

### The *Fab-7* element is required for stable chromatin contacts

The ability of 3D-CIS to induce TEI in Fab2L suggested that the primary role of the *Fab-7* element in the establishment of TEI is to mediate chromatin contacts between the transgenic and endogenous *Fab-7* elements. To determine whether induced chromatin contacts can trigger TEI at the Fab2L transgene even in the absence of the *Fab-7* element, we generated a new version of 3D-CIS in which the transgenic *Fab-7* was deleted (Figure S11A). As expected, phenotypically, this line resembled Fab2L-INS-PRE, with all individuals possessing uniform red eyes (Figures 1J and S11B). Similarly, this LacO-Fab2L-Fab7D line was unable to acquire a repressed epigenetic state by either selection or paramutation (Figures S11C–S11E). Activation of 3D-CIS by introduction of the LacI-TetR fusion protein was also unable to trigger TEI in this line (Figures S11F–S11I). However, FISH analysis revealed that chromatin contacts between the transgene and the endogenous *Fab-7* were not increased in this line. Indeed, in both the “OFF” and “ON” state, 3D-CIS-Fab7D flies did not show any significant contacts between the two loci, comparable with Fab2L; *Fab7[1]* (Figure S11J). This suggests that 3D-CIS is insufficient to mediate chromatin contacts on its own but rather acts to stabilize or reinforce contacts already established between the two *Fab-7* elements.

### Altered epigenetic states remain stable in the absence of artificially induced chromatin contacts

These results demonstrate a clear role for chromatin contacts in the initial triggering of TEI in Fab2L. However, due to experimental constraints, 3D-CIS remains active throughout the selection toward epilines. To determine whether the altered epigenetic states triggered by 3D-CIS can be maintained even in the absence of induced chromatin contacts, we crossed 3D-CIS epilines with a naive LacO-Fab2L. In the F2, flies lacking the TetR-LacI fusion protein (as determined by a GFP marker, see STAR Methods) were selected and counted. Even in the absence of the TetR-LacI inducing chromatin contacts, this F2 generation had a majority of individuals with primarily white eyes, in the case of the white epiline (Figure 5J), or primarily red eyes, in the case of the red epilines (Figure S12A). This parallels the results of paramutation when crossing with Fab2L epilines triggered by transient hemizygosity rather than 3D-CIS (Figures S12B and S12C). The LacO-Fab2L was therefore able to maintain the memory of its altered epigenetic state, even in the absence of artificially induced chromatin contacts with the endogenous *Fab-7*, demonstrating that enhancement of chromatin contacts is required to establish, but not maintain, TEI.

## Discussion

### GAF-mediated chromatin contacts and PRC2-mediated epigenetic variability together account for TEI at the Fab2L transgene

Our results highlight the crucial role played by two subdomains of the *Fab-7* element in the establishment of epigenetic variation at the Fab2L transgene and the maintenance of its memory across generations. These subdomains are an insulator and a PRE, which act through the recruitment of the transcription factors GAF and Pho. Alone, one of these regions remains sufficient to maintain a certain degree of variation and epigenetic memory at the Fab2L transgene, albeit in a manner skewed toward de-repression. Mutation of all GAF and Pho sites across both regions, however, eliminates all variation, demonstrating that at least some binding of these proteins is essential (Figures 1 and 2).

Our findings suggest that the insulator and PRE cooperate to control epigenetic regulation of the *Fab-7* element in two 686 Molecular Cell *85*, 677–691, February 20, 2025 ways. The first is the recruitment of PRC2, which deposits H3K27me3 in a stochastic manner, leading to a variable eye color phenotype. The second is to mediate chromatin contacts between the two distant *Fab-7* elements in the genome. However, mutation of these subdomains suggests that the PRE is the more important of the two regions for PRC2 recruitment (and thus epigenetic variability) (Figure 1E), whereas the insulator is much more involved in the establishment of chromatin contacts (Figure 4). Nevertheless, both elements contribute to some extent to both aspects of Fab2L regulation.

These differences in the effects of insulator and PRE mutation suggested differing roles for GAF and Pho, consistent with their known functions and the uneven distribution of their binding sites between the two domains. Indeed, the majority of GAF binding sites (6 out of 9) are located in the insulator, whereas most Pho sites (3 out of 4) are in the PRE (Figure 1B). Mutation of the GAF gene *Trl* confirmed this hypothesis, demonstrating that eliminating GAF functionality completely abrogates contacts (Figure 4). Based on these observations, we propose that GAF is the primary mediator of chromatin contacts between the Fab-7 elements, just as it has been shown to bridge other genomically distant but homologous genes,^42^ whereas Pho is the primary recruiter of PRC2 and thus responsible for the epigenetic variability at the Fab2L transgene. Together, these proteins thus mediate the dual functions of the *Fab-7* element, both of which are essential to TEI in this model system.

### PRC2-dependent epigenetic memory at the Fab2L locus

Our results point to a central role for chromatin contacts in the establishment of TEI at Fab2L, but not in the inheritance of alternative Fab2L gene expression across generations, as contacts are not present in early development (Figure S7). PRC2-deposited H3K27me3 is thus the primary epigenetic signal underpinning the variability at the Fab2L transgene and must be inherited by another mechanism independent of chromatin contacts.

Previous work has provided evidence of germline inheritance of H3K27me3 in *Drosophila*^45^ as well as other organisms.^46–48^ Another interesting possibility is raised by the discovery that PcG components on mitotic chromosomes in *Drosophila* can contribute to epigenetic memory across mitosis.^49^ That this mechanism could extend to meiosis is an interesting prospect, as it has been suggested that retention of DNA-bound proteins in development contributes to transgenerational epigenetic memory.^23^ PRC2, for example, which both binds to and deposits H3K27me3, has the potential to provide a positive feedback loop to stabilize transgenerational H3K27me3. In this way, inheritance of H3K27me3 both directly, by transmission through the germline, and indirectly, through stable polycomb protein binding, could provide redundancy and reinforcement to ensure a more reliable inheritance of epigenetic memory. Although our study is primarily concerned with explaining how TEI is triggered, future investigations into the mechanism by which this epigenetic state is transmitted across generations will provide a more complete picture of this instance of TEI.

### Chromatin contacts trigger PRC2-dependent TEI at the Fab2L locus

Our results indicate that the primary mechanism for the triggering of TEI at the Fab2L transgene is the promotion of physical contact within the nucleus between Fab2L and the endogenous *Fab-7*. Increased contact frequency can be induced by hemizygosity of *Fab-7* (Figure S2). One possibility for why this occurs is that in flies homozygous for *Fab-7*, the two homologous *Fab-7* loci preferentially contact each other, whereas when only one copy of *Fab-7* is present, it is free to form more stable contacts with the transgenic Fab2L. It would be interesting to test this possibility further.

We also demonstrated that synthetic chromatin contacts induced in our transgenic 3D-CIS also trigger TEI in the absence of any genetic perturbation (Figure 5). We note that this system is unable to mimic the effects of hemizygosity in the absence of an adjacent *Fab-7* element (Figure S10). This is explained by the fact that Fab2L-*Fab-7* contacts are already observed to a lesser extent in Fab2L; + individuals but are increased in Fab2L; *Fab [7]*/+ hemizygotes (Figure 4A). Thus, 3D-CIS acts to increase or stabilize the contacts already occurring between the two loci rather than driving the contacts *de novo*.

**Figure 6:**
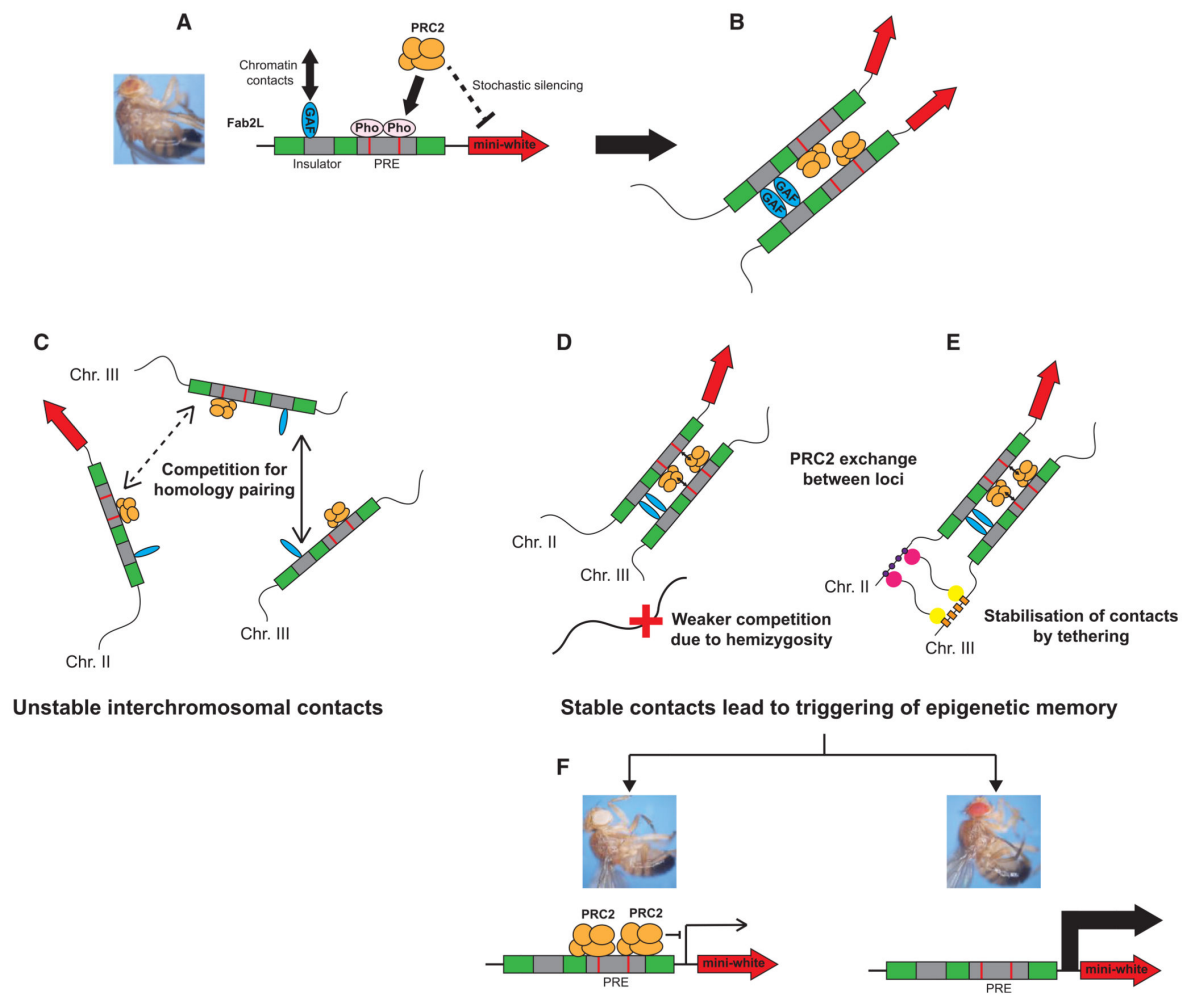
Model: Stabilization of interchromosomal contacts triggers an epigenetic memory of PRC2 binding (A) The *Fab-7* element recruits PRC2 by Pho binding to its PRE, leading to stochastic silencing of a *mini-white* transgene and a mosaic eye color. (B) This PRC2 recruitment is coupled with chromatin contacts with other *Fab-7* elements mediated by GAF through an insulator region. (C) When more than one copy of *Fab-7* is present in the genome, contacts can be initiated between distant *Fab-7* elements, but these contacts are outcompeted by inter-allelic contacts and remain transient. (D and E) Stabilization of these contacts can be achieved through hemizygosity of one *Fab-7* copy (D) or through synthetic biology tools (E). This stabilization leads to the exchange of PRC2 between the PREs, resulting in either increased PRC2 association and silencing or decreased PRC2 association and de-repression and triggering an epigenetic memory of this altered association. (F) Over generations, these slight differences can be selected to extremes, resulting in either very strong or very weak repression and strikingly different phenotypes.

These data can be integrated to provide a model for how TEI is established and maintained. Importantly, any mechanism explaining how this trigger occurs must also explain why either repression or de-repression can be triggered. We therefore propose a model whereby stabilization of Fab2L-*Fab-7* chromatin contacts allows for the exchange of PRC2 between the endogenous and transgenic *Fab-7* elements, thereby triggering an epigenetic memory that can be selected toward extremes over generations (Figure 6).

We propose that, in naive Fab2L flies, PRC2 is stochastically recruited to the transgene by Pho, leading to a random mosaic eye color (Figure 6A). Chromatin contacts are mediated by GAF but, in the absence of manipulation, these contacts primarily occur between homologous alleles, i.e., Fab2L to Fab2L or endogenous *Fab-7* to endogenous *Fab-7* (Figure 6B). Although interchromosomal contacts between Fab2L and *Fab-7* do occasionally occur, they are transient and outcompeted by the preferential interaction between homologous alleles that is common in dipteran species^50^ (Figure 6C). Stabilization of these contacts is achieved upon *Fab-7* hemizygosity (Figure 6D) because the remaining endogenous *Fab-7*, having lost its preferred interaction partner, is free to form more stable contacts with its imperfect transgenic partner without being outcompeted by its homologous allele. This situation can be mimicked in a homozygous state and in the absence of genetic perturbation, thanks to the 3D-CIS transgenic system, which artificially stimulates chromatin contacts between Fab2L and *Fab-7*, making them interact preferentially with each other rather than with their homologous alleles (Figure 6E).

A recent perspective has considered the implications of the classic “source-sink” model of epigenetics in the chromatin age.^51^ In this model, different “sinks” (in our case: Pho binding sites) compete to be filled by a limited “source” (binding of PRC2). Although many PRC2 complexes are present in the nucleus, and the two *Fab-7* elements are unlikely to influence each other when they are physically distant, we propose that the bringing together of the endogenous and transgenic *Fab-7* elements puts them in direct competition for the binding of PRC2 in the local environment. PRC2 thus becomes a limited source that is exchanged between the binding sites with the PREs of the transgenic and endogenous *Fab-7* elements.

The endogenous *Fab-7* is unlikely to be affected by this competition in the long term. Indeed, in its natural genomic context, within the highly regulated BXC locus, it is subject to much more fine-tuning from the many regulatory mechanisms that ensure the proper expression of the crucial Hox genes within the cluster. Once separated from the transgenic locus, any discrepancy in PRC2 binding is thus likely to be corrected. The transgenic *Fab-7*, on the other hand, divorced from its genomic context, may be much more susceptible to perturbations in PRC2 binding. Thus, an epigenetic memory of altered PRC2 binding in early development may persist into adulthood and even to the next generation. Although these inherited epigenetic differences may initially be small, and are indeed essentially indistinguishable in the first generation of our experiments, repeated selection could eventually lead to substantial differences, with each generation resulting in an incremental change in the number of ommatidia either repressed or de-repressed, ultimately resulting in fully monochrome eye color (Figure 6F).

A few aspects of this model are worth highlighting. First, it would predict that a Fab2L/+; *Fab7[1]*/+ double hemizygote, in which both loci have lost their preferential interaction partner on the homolog, should be an even more effective trigger of TEI than single hemizygosity. Although we have not examined this in detail, our different TEI-triggering crosses support this, as epilines derived from Fab2L/+; *Fab7[1]*/+ double hemizygotes tend to reach fixation faster than those derived from Fab2L; *Fab7 [1]*/+ single hemizygotes during selection (Figure S2). Second, exchange of PRC2 binding between Fab2L alleles rather than between Fab2L and *Fab-7*, could also explain how paramutation is able to transfer epigenetic state, albeit imperfectly, between homologous alleles. Our model thus accounts for both the triggering of epiallelic identity, and its horizontal transfer by paramutation.

### A broader role for hemizygosity and chromatin contacts in triggering TEI

One major question in the field of epigenetic inheritance is how heritable epigenetic variability, or epimutation, arises in the first place. Studies in plants suggest that heritable changes in DNA methylation can occur apparently spontaneously in these organisms, leading to long-term epigenetic differences between lines.^52–54^ Recent studies in *Caenorhabditis elegans* have extended these observations to metazoans and to RNA and chromatin-based epigenetic changes.^55,56^ Other studies have sought to identify environmental triggers for TEI, directly linking epigenetic variation to an external stress to which it is intended to respond.^2,57,58^ The final prominent candidate for sources of epigenetic variation is genetic perturbation, different types of which have been shown to trigger TEI in a variety of organisms.^15,16,59,60^

In the *Drosophila* Fab2L line, this genetic perturbation takes the form of transient hemizygosity of the endogenous *Fab-7* region for at least one generation. It is interesting to note that, unlike some cases of genetically triggered TEI, in Fab2L the epigenetic memory is triggered and maintained not at the locus that is perturbed (the endogenous *Fab-7*) but elsewhere in the genome (the Fab2L transgene), testifying to the ability of *trans*-interactions to induce TEI. *Fab-7* hemizygosity has been found to have a similar effect on another PRE-containing gene in the distant Antennapedia cluster, with a phenotype that persisted for several generations after restoration of *Fab-7* homozygosity.^19^ This raises the question of whether similar mechanisms could be acting to trigger TEI at other loci in natural populations.

Recent sequencing of wild *Drosophila melanogaster* lines has revealed the incredible genomic variation between populations of this single species. This includes numerous and large-scale deletions, duplications, and translocations across the genome.^61^ Mixing of two such genomically disparate populations would lead to a number of hemizygosity or heterozygosity events, as well as homology between very distant loci reminiscent of what is observed in Fab2L. Breeding in the wild thus has the potential to lead to many instances of naturally occurring genetic perturbation as potential triggers for TEI. Just as in Fab2L, it could be that establishment of TEI might be possible only between certain regions that are already prone to contact each other. In this respect, PRC2 targets may be particularly interesting candidates for naturally occurring TEI. Indeed, as previously mentioned, many PRC2 targets are frequently clustered within the nucleus in polycomb bodies, forming a large domain of silenced chromatin.^41^ Interestingly, genes regulated in this manner are more likely to possess both an insulator region and a PRE, just like *Fab-7*. Our study provides insight into the mechanisms by which this type of epimutation could occur, but it is only by extending this insight to a broader context that we will be able to determine the role of TEI in the phenotypic variation—and thus potentially the adaptation—of natural populations.

### Limitations of the study

We used the Fab2L line to investigate the role of chromatin contacts in *Drosophila* epigenetic inheritance, allowing us to perform genetic manipulations and selection experiments without disruption of endogenous sequences or effects on the organisms’ fitness. Although these advantages were essential in arriving at our mechanistic model, further work is required to determine the broader relevance of this model at other endogenous loci and in more natural conditions. To better determine the role of GAF, we utilized the *Trl^R85^* loss-of-function mutant, which causes lethality at the larval stage, thus preventing us from fully investigating the effect of GAF disruption in the adult fly and across generations, to determine, for instance, whether mutation of GAF prevents triggering of epigenetic inheritance or influences adult eye color. We devised the 3D-CIS synthetic biology system to induce chromatin contacts between loci by expression of a transgenic protein construct. Expression of this construct is constitutive in the fly, occurring in all tissues and all developmental stages. This does not reflect the likely biological scenario in which contacts between the two loci may only be required briefly and at a key stage in development to induce epigenetic inheritance. Further work with more controlled expression could help to refine the model to more precisely indicate the window during which these contacts are required. 3D-CIS is also unable to induce contacts between the loci in the absence of a *Fab-7* element, suggesting that it acts to reinforce or stabilize contacts already present. This regrettably means that it is not possible to use the system to investigate the impact of induced chromatin contacts divorced from any influence of the *Fab-7* element.

## Resource Availability

### Lead contact

Further information and requests for resources and reagents should be directed to the lead contact, Giacomo Cavalli (giacomo.cavalli@igh.cnrs.fr).

### Materials availability

Unique materials generated by this study, including transgenic fly lines, are available upon request from the lead contact.

## Star⋆Methods

### Key Resources Table

**Table:** 

REAGENT or RESOURCE	SOURCE	IDENTIFIER
Antibodies		
H3K27me3	Active Motif	Cat#39155; RRID: AB_2561020
E(z)	Ogiyama et al.^39^	N/A
GAF	Schuettengruber et al.^62^	N/A
Pho	Brown et al.^63^	N/A
GFP	Abcam	Cat#ab290; RRID: AB_303395
Rabbit IgG	Cell Signalling	Cat#2729; RRID: AB_1031062
Chemicals, peptides, and recombinant proteins		
EDTA-free protease inhibitor cocktail	Roche	Cat#11873580001
Paraformaldehyde	Thermo Scientific	Cat#28906
Proteinase K	Thermo Scientific	Cat#EO0491
Spermidine	Thermo Scientific	Cat#A19096.22
Spermine	Thermo Scientific	Cat#132750010
Deionised Formamide	Sigma Aldrich	Cat#S4117
DAPI	Sigma Aldrich	Cat#10236276001
Prolong Gold Antifade	Invitrogen	Cat#P36930
Critical commercial assays		
Protein A Dynabeads	Invitrogen	Cat#10002D
Light Cycler 480 SYBR green I Master mix	Roche	Cat#04887352001
FISH tag DNA kit	Invitrogen	Cat#F32951
Deposited data		
Microscopy images 1	Mendeley Data	https://doi.org/10.17632/8rz25rrtzj.1
Microscopy images 2	Mendeley Data	https://doi.org/10.17632/mg2rgpjp23.1
Microscopy images 3	Mendeley Data	https://doi.org/10.17632/yd27df555b.1
Microscopy images 4	Mendeley Data	https://doi.org/10.17632/h38kgb5zkf.1
Experimental models: Organisms/strains		
Fab2L	Bantignies et al.^25^	N/A
Fab2L; *Fab7[1]*	Bantignies et al.^25^	N/A
Fab2L-R*	Ciabrelli et al.^19^	N/A
Fab2L-W*	Ciabrelli et al.^19^	N/A
w[1118]	BDSC	RRID: BDSC_5905
37B-AttP	This paper	N/A
Fab2L-INS	This paper	N/A
Fab2L-PRE	This paper	N/A
Fab2L-INS-PRE	This paper	N/A
2A3-AttP	BDSC	RRID: BDSC_24480
21xLacO-Fab2L	This paper	N/A
7xTetO-Fab7	This paper	N/A
TetR-GFP-LacI	This paper	N/A
TetR-GFP-TetR	This paper	N/A
LacI-GFP-LacI	This paper	N/A
FabL, *black[1]*	Ciabrelli et al.^19^	N/A
Fab2L-INS, *black[1*	This paper	N/A
Fab2L-PRE, *black[1*	This paper	N/A
Fab2L-INS-PRE, *black[1*	This paper	N/A
Fab2L-*Fab-7*Δ	This paper	N/A
Fab2L-*Fab-7*Δ, *black[1]*	This paper	N/A
w[1118]; *black[1]*	BDSC	RRID: BDSC_227
TM3, Sb, Kr-GFP	Casso et al.^64^	RRID: BDSC_5195
Fab2L, *black[1]; TrlR85/TM6*	Ciabrelli et al.^19^	N/A
Fab2L; TrlR85/TKG	This paper	N/A
Fab2L; TrlR85, Fab*7[1]*/TKG	This paper	N/A
Oligonucleotides		
37B FISH Probe 1 F: TGCACTTGACCACGACTTTG	Ciabrelli et al.^19^	N/A
37B FISH Probe 1 R: CCTGCTCGAATGGAAGGGTA	Ciabrelli et al.^19^	N/A
37B FISH Probe2 F: AGTGCGCCAAACATAAGTCC	Ciabrelli et al.^19^	N/A
37B FISH Probe 2 R: GCTTTGTACAGACTGGTGGC	Ciabrelli et al.^19^	N/A
37B FISH Probe 3 F: TTCAAGATCGGGGCCTCTTT	Ciabrelli et al.^19^	N/A
37B FISH Probe 3 R: CCTCAAAAGTGACGCCTTCC	Ciabrelli et al.^19^	N/A
37B FISH Probe 4 F: AGACTCGTTACGCCTTTGGA	Ciabrelli et al.^19^	N/A
37B FISH Probe 4 R: ATTGCTCCCTTTGGCACTTG	Ciabrelli et al.^19^	N/A
37B FISH Probe 5 F: AAACCGAGTGCTATGGTCCA	Ciabrelli et al.^19^	N/A
37B FISH Probe 5 R: TCTTGAGCTCTTCCAGGGTG	Ciabrelli et al.^19^	N/A
37B FISH Probe 6 F: ACGACGACAAGCCCTAGAAA	Ciabrelli et al.^19^	N/A
37B FISH Probe 6 R: CCAAATGCCCTTCCAAACCA	Ciabrelli et al.^19^	N/A
89E FISH Probe 1 F: TCCCTTGCTTCGAAGGAGAG	Ciabrelli et al.^19^	N/A
89E FISH Probe 1 R: ATGTGTGTGTTGGCTGGTTG	Ciabrelli et al.^19^	N/A
89E FISH Probe 2 F: ATTCCAACCACCCATTTGCC	Ciabrelli et al.^19^	N/A
89E FISH Probe 2 R: CCTCTCTCTTGGCCAACTCA	Ciabrelli et al.^19^	N/A
89E FISH Probe 3 F: CAAGGGTCACGCTGAAACAA	Ciabrelli et al.^19^	N/A
89E FISH Probe 3 R: GCGAATATGTGAGTGCTGCA	Ciabrelli et al.^19^	N/A
89E FISH Probe 4 F: GGGAGGGAGAACGGGTTTAT	Ciabrelli et al.^19^	N/A
89E FISH Probe 4 R: CCGTTCCGCTGCTAAATCAA	Ciabrelli et al.^19^	N/A
89E FISH Probe 5 F: GCTTGAGTCGTTAAGAGGCG	Ciabrelli et al.^19^	N/A
89E FISH Probe 5 R: CCTTCTTGCTCGCCTGAATC	Ciabrelli et al.^19^	N/A
89E FISH Probe 6 F: TATCCAGCTACCCAACCACC	Ciabrelli et al.^19^	N/A
89E FISH Probe 6 R: TGTGGCTTTTACGAGGTCCT	Ciabrelli et al.^19^	N/A
Fab2L insulator qPCR primer F: AAGAGCGTCCGCTCACTAAC	This paper	N/A
Fab2L insulator (Wild-type) qPCR primer R: CAAACCTAGCCGCTCTCTTG	This paper	N/A
Fab2L insulator (mutant) qPCR primer R: CAAACCTAGCCGCAGTGTTG	This paper	N/A
Fab2L PRE (Wild-type) qPCR primer F: TCATTTTCAGCTCGGCCATC	This paper	N/A
Fab2L PRE (mutant) qPCR primer F: TCATTTTCAGCTCGGTGCAC	This paper	N/A
Fab2L PRE qPCR primer R: TTTTCAGCCCCGAAAATGCC	This paper	N/A
Fab2L LacZ qPCR primer F: TGCAGGATATCCTGCTGATG	This paper	N/A
Fab2L LacZ qPCR primer R: TTGGCTTCATCCACCACATA	This paper	N/A
Fab2L mini-W qPCR primer F: CGCTTCTGATCTGCGATGAG	This paper	N/A
Fab2L mini-W qPCR primer R: CTGGGAGTGCCCAAGAAA	This paper	N/A
37B A qPCR primer F: CTGCGCAACACGAAATTAGA	This paper	N/A
37B A qPCR primer R: CCTTGTTTCCGGGTACTCAA	This paper	N/A
37B B qPCR primer F: GAGCGCGCACTTAATCGAC	This paper	N/A
37B B qPCR primer R: TGCGGTTAAGGGCCAATTTT	This paper	N/A
89E A qPCR primer F: TTTCTCCACTTCCGACGAAC	This paper	N/A
89E A qPCR primer R: CGGTTCCATTCTCAACTCGT	This paper	N/A
89E B qPCR primer F: CTCTCCGTTTCCCTTTAGGC	This paper	N/A
89E B qPCR primer R: GCTGGTTGTGCAGGTGAATA	This paper	N/A
LacO A qPCR primer F: TAGCATGTCCGTGGGGTTT	This paper	N/A
LacO A qPCR primer R: ggacGAATTCAGCTCCCAAC	This paper	N/A
LacO B qPCR primer F: tgcacacctgcgatcataac	This paper	N/A
LacO B qPCR primer R: AGTCCACAAAGCTTGGATCC	This paper	N/A
TetO A qPCR primer F: GGCAGTGGGAAGTCGTATTT	This paper	N/A
TetO A qPCR primer R: gaattCCACCGGCTTCTGTA	This paper	N/A
TetO B qPCR primer F: tgcacacctgcgatcataac	This paper	N/A
TetO B qPCR primer R: TTCTGGGGCTTTACCGTTG	This paper	N/A
Fab7 A qPCR primer F: TGTTTATTCATCGCCTTTTGC	This paper	N/A
Fab7 A qPCR primer R: TTTAGCCCTGCGAAGATATG	This paper	N/A
Fab7 B qPCR primer F: AAAGAAACCCATTGGTGCAG	This paper	N/A
Fab7 B qPCR primer R: CAAAGTTGGATGCATTGTGG	This paper	N/A
Fab7 C qPCR primer F: AAGTGCAGCGCCCAATAAG	This paper	N/A
Fab7 C qPCR primer R: GCGACATTCTACCTCGCTCT	This paper	N/A
Recombinant DNA		
pCFD3-dU6:3gRNA	Addgene	Cat#49410
pDsRed-AttP	Addgene	Cat#51019
pAct-FRT-stop-FRT3-FRT-FRT3-Gal4 attB	Addgene	Cat#52889
FU-TetO-Gateway	Addgene	Cat#43914
pCL-CTIG	Addgene	Cat#14901
pKG215	Addgene	Cat#45110
pHD-DsRed	Addgene	Cat#51434
pUC-TALO8	Addgene	Cat#83409
pHD-AttB-Fab2L-INS-LoxP-DsRed	This Paper	N/A
pHD-AttB-tTA-EGFP-LacI-LoxP-DsRed	This Paper	N/A
pHD-AttB-tTA-EGFP-tTA-LoxP-DsRed	This Paper	N/A
pHD-AttB-LacI-EGFP-LacI-LoxP-DsRed	This Paper	N/A
pHD-7xTetO-Fab7	This Paper	N/A
pHD-21xLacO-Fab2L	This Paper	N/A
Software and algorithms		
Imaris software	Oxford Instruments	N/A

### Experimental Model and Study Participant Details

#### Fly stocks and culture

Flies were raised in standard cornmeal yeast extract media. Standard temperature was 21°C, with the exception of P0 and F1 crosses in the experiments of Fab2L epiallele establishment by hemizygosity or 3D-CIS (Figures 2, 5F, 5G, S2, S7A–S7D, S9D, S9E, and S10D–S10I), for which temperature was 18°C. The Fab2L and Fab2L; *Fab7[1]* lines were described in Bantignies et al.^25^ The Fab2L, *black[1]* line and pre-established Fab2L epilines (Fab2L-R* and Fab2L-W*) were described in Ciabrelli et al.^19^

For generation of the Fab2L mutant lines, a transgenic line was made containing an AttP insertion site at cytogenetic position 37B, by CRISPR-Cas9 of a w[1118] line (Bloomington Drosophila Stock Center) to cut at the exact site of Fab2L transgene insertion in the Fab2L line. Fab2L-INS was then generated by Phi-recombination of an AttB-containing plasmid (cloned from Addgene plasmid 52889) containing the entirety of the Fab2L transgene, with directed mutations of the insulator GAF and Pho sites, into this 37B-AttP line. Injection services for these two lines were provided by BestGene Inc. Fab2L-PRE and Fab2L-INS-PRE were then generated by CRISPR-Cas9 editing of Fab2L-INS, with a two-guide RNA strategy designed and implemented by Rainbowgene Transgenic Flies Inc.

To create the 3D-CIS system, arrays of 7 Tet operators (cloned from Addgene plasmid 43914) and 21 Lac operators (cloned from Addgene plasmid 83409) were inserted adjacent to the endogenous or transgenic *Fab-7*, respectively, using a single guide RNA to cut immediately to their 3′. Cassettes encoding recombinant proteins combining the Lac and/or Tet repressors marker (cloned from Addgene plasmids 45110 and 14910, respectively) with a GFP marker (cloned from Addgene plasmid 45110) under expression of an *Actin-5C* promoter marker (cloned from Addgene plasmid 52889) were inserted into chromosome arm 3L separately by Phi recombination into an established AttP containing line (Bloomington 24480) to generate TetR-GFP-LacI, TetR-GFP-TetR and LacI-GFP-LacI lines. The transgenes encoding these proteins were then recombined with the TetO-*Fab-7*, and introduced into a Fab2L background, ready to be crossed with the LacO-Fab2L as described in Figure S7. All injections for these lines were provided by BestGene Inc. The Fab2L-*Fab-7*D line was derived from the LacO-Fab2L line by CRISPR-Cas9 targeted deletion of the transgenic *Fab-7*, designed and implemented by Rainbowgene Transgenic Flies Inc.

Fab2L-INS, *black[1]*, Fab2L-PRE, *black[1]*, Fab2L-INS-PRE, *black[1]* and Fab2L-Fab7D, *black[1]* were generated by recombining the Fab2L transgene with the *black[1]* allele from the w[1118]; *black[1]* line (Bloomington Drosophila Stock Center). Fab2L flies containing the *Trl^R85^* mutant balanced on the TM3, Sb, Kr-GFP (TKG) balancer chromosome, described in Casso et al.,^64^ were obtained by crossing Fab2L, *black[1]*; *Trl^R85^*/TM6 flies (from Ciabrelli et al.^19^) with *Pc*/TKG flies, and the *Trl^R85^* mutant was combined with *Fab7 [1]* by recombination to obtain a Fab2L; *Trl^R85^*, *Fab7[1]*/TKG line. The TKG balancer contains a fluorescent GFP marker expressed under control of the *krüppel* promoter, giving a distinct GFP pattern in mid to late embryos, thus allowing for selection of Fab2L; *Trl^R85^* homozygous embryos (see below).

### Method Details

#### Chromatin Immunoprecipitation and antibodies

0 to 16 hour old embryos were collected in Embryo Wash Buffer (0.03% Triton X-100, 140mM NaCl) and dechorionated with bleach. Samples were crosslinked in 1 ml A1 buffer (60 mM KCl, 15 mM NaCl, 15 mM HEPES [pH 7.6], 4 mM MgCl2, 0.5% Triton X-100, 0.5 mM dithiothreitol (DTT), 10 mM sodium butyrate and complete EDTA-free protease inhibitor cocktail [Roche]), in the presence of 1.8% formaldehyde. Samples were homogenized with a micropestle and incubated for a total time of 15 minutes at room temperature. Crosslinking was stopped by adding 350 mM glycine followed by incubation for 5 min. The homogenate was transferred to a 2 ml tube and centrifuged for 5 minutes, 4,000g at 4°C. The supernatant was discarded, and the nuclear pellet was washed three times in 2 ml A1 buffer and once in 2 ml of Lysis buffer (140 mM NaCl, 15 mM HEPES [pH 7.6], 1 mM EDTA, 0.5mM EGTA, 1%Triton X-100, 0.5mMDTT, 0.1% sodium deoxycholate, 10 mM sodium butyrate and complete EDTA-free protease inhibitor cocktail [Roche]) at 4°C. Nuclei were than resuspended in 1.5 ml Lysis buffer in the presence of 0.1% SDS and 0.5% N-Laurosylsarcosine, transferred to a 15 ml falcon tube and incubated for 2 hours with agitation at 4°C. Samples were adjusted to 3 ml and chromatin was sonicated using a Q700 sonicator with microtip (QSonica) for a total of 6 minutes and 30 seconds at amplitude 50 (settings: 30 s on, 1min 30 s off x 13 cycles) in an ice bucket. Sheared chromatin had size range of 100 to 300 base pairs. After sonication and 5 minutes high-speed centrifugation at 4°C, fragmented chromatin was recovered in the supernatant and aliquoted in 5 mg (for H3K27me3 ChIP) or 20 mg (for non-histone protein ChIP) aliquots adjusted to a volume of 500 ml in Lysis Buffer with 0.1% SDS and 0.5% N-Laurosylsarcosine for storage at -20°C.

To perform the ChIP, samples were thawed on ice and chromatin was precleared by addition of 15 ml of Protein A Dynabeads (Invitrogen 10002D) followed by incubation for at least 1 hour at 4°C. Dynabeads were removed on a magnetic rack and antibodies were added at a dilution of 1:100 (a mock control in the presence of rabbit IgG was performed at the same time, while an input of the same size was set aside). Samples were incubated for overnight at 4°C on a rotating wheel. 30 ml of Protein A Dynabeads were added and incubation was continued for at least 2 hours at 4°C. Antibody-protein complexes bound to beads were washed 4 times in Lysis Buffer with 0.05% SDS and twice in TE Buffer (0.1 mM EDTA, 10 mM Tris (pH 8)) in 1 ml each time. Chromatin was eluted from beads in 100 μl of 10 mM EDTA, 1% SDS, 50 mM Tris (pH 8) at 65°C for 15 minutes and eluted again in 150 μl of 10 mM EDTA, 0.67% SDS, 50 mM Tris (pH 8) at 65°C for 15 minutes, with the eluate collected on a magnetic rack each time. The 250 μl eluates and 250 μl of the Input DNA samples (1:2 input) were incubated overnight at 65°C to reverse crosslinks and treated with Proteinase K for 3 hours at 56°C. DNA was isolated by addition of an equal volume of phenol-chloroform, supernatants collected and then ethanol precipitated for 2 hours at -20°C in the presence of 20 μg glycogen by addition of 25 μl 3M sodium acetate and 625 μl ethanol. Samples were centrifuged at high speed for 1 hour and washed in 500 μl of 70% ethanol before resuspension in 200 μl H2O. Immunoprecipitated DNA was used to analyze the enrichment of specific DNA fragments by real-time PCR (qPCR), using a Roche Light Cycler 480 and the Light Cycler 480 SYBR green I Master mix. For each amplicon, IP DNA was normalized to Input DNA. The ChIP/Input ratio was further normalized to a positive control region (engrailed). ChIP amplicons for the insulator or PRE regions were specific to either the WT or mutated transgenic sequence, depending on the genotype analysed. Antibodies used in this study were as follows: anti-GAF polyclonal antibody^62^; anti-Pho polyclonal antibody^62,63^; anti-E(z) polyclonal antibody^39^; anti-H3K27me3 polyclonal antibody (Active Motif 39155), anti-GFP polyclonal antibody (Abcam ab290), normal rabbit IgG (Cell Signalling 2729).

#### Fluorescence in situ hybridization

Two-color 3D FISH was performed as previously described.^65^ For a detailed protocol, see Bantignies and Cavalli.^66^ Briefly, embryos were collected and washed in dH2O before dechorionation with bleach. Most embryos were collected at stage 14-15 after maturation for 14.5 to 18.5 hours at 21°C. For other stages maturation was for: 2-4 hours at 21 (stage 4-5), 4.5-6 hours at 21 (stage 8-9), 6-9 hours at 21 (stage 10-11) and 15.5-20 hours at 18 (stage 12-13). Fab2L; *Trl^R85^* homozygous embryos were obtained by sorting for GFP negative embryos after dechorionation. Embryos were then fixed in buffer A (60 mM KCl; 15 mM NaCl; 0.5 mM spermidine; 0.15 mM spermine; 2 mM EDTA; 0.5 mM EGTA; 15 mM PIPES, pH 7.4) with 4% paraformaldehyde for 25 min in the presence of heptane then devitellinized by adding methanol to the heptane phase, extracted and washed three times in methanol. Embryos were kept for a maximum of 4 months in methanol at 4C before proceeding to FISH. Fixed embryos were sequentially re-hydrated in PBT (PBS, 0.1% Tween 20) before being treated with 100–200 μg/ml RNaseA in PBT for 2 hours at room temperature. Embryos were then sequentially transferred into a pre-Hybridization Mixture (pHM: 50% formamide; 4XSSC; 100 mM NaH2PO4, pH 7.0; 0.1% Tween 20). Embryonic DNA was denatured in pHM at 80°C for 15 minutes. The pHM was removed, and denatured probes diluted in the FISH Hybridization Buffer (FHB: 10% dextransulfat; 50% deionized formamide; 2XSSC; 0.5 mg/ml Salmon Sperm DNA) were added to the tissues without prior cooling. Hybridization was performed at 37°C overnight with gentle agitation. Post-hybridization washes were performed, starting with 50% formamide, 2XSSC, 0.3% CHAPS and sequentially returning to PBT. After an additional wash in PBS-Tr, DNA was counterstained with DAPI (at a final concentration of 0.1 ng/μl) in PBT and embryos were mounted with ProLong Gold Antifade (Invitrogen).

FISH probes for the 37B and 89E regions were made from a previous design described in Ciabrelli et al.^19^ For each region, 6 non-overlapping probes of between 1.2 and 1.7kb covering an area of approximately 12kb were generated using the FISH Tag DNA kit with Alexa Fluor 555 or Alexa Fluor 647 dyes (Invitrogen Life Technologies). 100ng of each probe were added to the 30μL of FHB for hybridization.

#### Microscopy and image analysis

For the FISH, the 3D distances between 37B and 89E loci were acquired and measured as follows: due to somatic pairing of homologous chromosomes in *Drosophila*, the majority of the nuclei in embryos show a single FISH spot for each probe. In the cases of non-overlap FISH signals between homologues, the closest distance between the centres of the two probes was considered. To measure distances, 3D stacks were collected from 3-5 different embryos. Optical sections were collected at 0.5 μm intervals along Z-axis using a Leica SP8-UV microscope, Montpellier Resources Imaging (MRI) facility, or a Zeiss AXIO imager, Oxford Micron facility. Relative 3D distances between FISH signals were analyzed in approximately 80 to 120 nuclei per 3D stack using the Imaris software (Oxford Instruments). The distance distribution between the two probes was obtained by pooling replicates for each condition.

### Quantification and Statistical Analysis

#### Statistical testing

To test for differences in the distribution of eye colour between fly populations we used Fisher’s exact test (for paramutation crosses and fly selection experiments done in a single line) or the two-tailed student’s *t*-test in cases where percentages were averaged across several repeats (selection experiments done in several lines). FISH distance measurements were compared by ANOVA. For average distance distributions, we used a two-way ANOVA, taking into account the embryo in which each measurement was taken to ensure that there were no significant differences between embryos of the same genotype/stage. Average numbers of paired loci were compared by one-way ANOVA. ChIP signal levels were compared using the two-tailed student’s *t*-test.

## Data Availability

Microscopy data have been deposited at Mendeley Data and are publicly available as of the date of publication at https://doi.org/10.17632/8rz25rrtzj.1, https://doi.org/10.17632/mg2rgpjp23.1, https://doi.org/10.17632/yd27df555b.1, and https://doi.org/10.17632/h38kgb5zkf.1. All data reported in this paper will be shared by the lead contact upon request. This paper does not report original code. Any additional information required to reanalyze the data reported in this paper is available from the lead contact upon request.

## Highlights

- Transgenerational epigenetic inheritance (TEI) of H3K27me3 occurs in Drosophila
- Both polycomb repressive complex 2 and GAGA factor contribute to TEI of H3K27me3
- Chromatin contacts mediated by GAGA factor are required to trigger this TEI
- Artificially inducing chromatin contacts in vivo is sufficient to trigger TEI

## In brief

In some cases, non-genetic information can be inherited across generations. Fitz-James et al. investigate the mechanism by which such epigenetic information becomes heritable in fruit flies. They show that physical contact between regions of DNA within the cell is able to trigger the histone modification H3K27me3 to become heritable.

## Footnotes

**Supplemental information**
Supplemental information can be found online at https://doi.org/10.1016/j.molcel.2024.11.021.
